# Sea Buckthorn Pretreatment, Drying, and Processing of High-Quality Products: Current Status and Trends

**DOI:** 10.3390/foods12234255

**Published:** 2023-11-24

**Authors:** Xuetao Zhang, Mengqing Li, Lichun Zhu, Zhihua Geng, Xinyu Liu, Zheyu Cheng, Mengxu Zhao, Qian Zhang, Xuhai Yang

**Affiliations:** 1 College of Mechanical and Electrical Engineering, Shihezi University, Shihezi 832003, China; 2 Engineering Research Center for Production Mechanization of Oasis Special Economic Crop, Ministry of Education, Shihezi 832003, China; 3 Xinjiang Production and Construction Corps, Key Laboratory of Modern Agricultural Machinery, Shihezi 832003, China

**Keywords:** sea buckthorn, drying kinetics, drying quality, high-quality process, research progress

## Abstract

Sea buckthorn is a kind of berry rich in nutritional and industrial value. Due to its thin skin, juicy pulp, and short shelf life, it is usually preserved via freezing methods or directly processed into sea buckthorn puree after harvest. It can also be dried and processed into products such as dried sea buckthorn fruit, freeze-dried sea buckthorn powder, and sea buckthorn oil. This review, therefore, provides an overview of the existing state of drying and high-quality processing of sea buckthorn. The effects of different pretreatment and drying techniques on the drying characteristics and quality of sea buckthorn and the existing problems of superior-quality processing of sea buckthorn products are summarised. The development trend of sea buckthorn drying methods and the ways to achieve high-quality processing of sea buckthorn products are indicated. These ways are mainly related to the following: (1) The application of combined pretreatment and drying techniques to find a balance between economy, ecology, and efficiency; (2) Introducing new online measurement and control technology into drying equipment; (3) Optimising the existing process to form a complete sea buckthorn industrial chain and develop the sea buckthorn deep-processing industry.

## 1. Introduction

With the *Action Plan of the United Nations Decade on Ecosystem Restoration*, the global promotion of returning farmland to forests and afforesting barren mountains is having far-reaching socio-environmental effects [1,2]. Sea buckthorn (*Hippophae rhamnoides* L.) is a deciduous shrub belonging to the genus sea buckthorn in the family Elaeagnaceae [3]. Sea buckthorn is a berry plant with the unique ability to endure both drought and cold conditions [4]. In addition, it can be used as a windbreak, fixing sand and nitrogen to promote the growth of other plants [5,6,7,8]. Sea buckthorn is known worldwide as a medicinal and food plant that is enriched with vitamins, flavonoids, and polyphenols. Therefore, it has substantial economic benefits and development potential [9,10,11,12,13,14]. Various products can be processed from sea buckthorn. Not only can it be used in making dried sea buckthorn pulp [15], sea buckthorn beverage [16], and sea buckthorn freeze-dried powder [17] but it can also be made into sea buckthorn wine [18] and sea buckthorn oil [19], among other products. Sea buckthorn oil contains a number of pharmacological properties, including the ability to protect cardiovascular and cerebral vessels, improve circulation, and boost immunity. And, it has numerous active components, one of which, SOD (superoxide dismutase), is a key natural beauty ingredient [20,21,22].

Sea buckthorn pulp will ferment and deteriorate at room temperature for a short period of time due to its high water content of 80–90%, and the thin skin of the pulp is prone to rupture. As a consequence, it lacks the ability to store and the convenience of transporting water [23]. That leads to its short shelf life after harvesting. If the drying of sea buckthorn is not performed in a timely manner, it is extremely susceptible to microbial contamination and spoilage. Currently, sea buckthorn is commonly preserved using freezing methods before being processed [24]. However, the nutritional value of sea buckthorn decreases with prolonged freezing time [25]. Another method of preservation is to produce sea buckthorn beverages directly after harvesting them [26]. Drying is an efficient processing method to extend the shelf life of berry products. It is advantageous for transportation and storage. Moisture is swiftly removed from the material, but a considerable amount of nutrients and active substances is still retained during drying [27,28,29]. Sea buckthorn pulp, after vacuum freeze-drying, can be brewed, compounded, and fermented to make juice, wine, and other products. Hence, the drying of sea buckthorn pulp after harvesting is a crucial step in the process.

Agricultural product drying is a complicated, non-stationary heat and mass transport process. It is a heat and mass transfer process that occurs between the substance and the external medium [30,31]. At present, the traditional drying techniques for sea buckthorn include natural drying and hot-air drying [32,33]. Natural drying uses solar or wind energy to evaporate the internal moisture of materials. Hot-air drying works by heating the air with thermal energy to evaporate water inside the material. However, two traditional drying methods are inefficient and have terrible drying quality. Moreover, the wasted heat from hot-air drying will have a negative impact on the environment [34]. Therefore, the primary purpose of contemporary research on new drying technology is focused on reducing drying energy consumption, cost, and other issues [35] to improve drying quality and efficiency. New drying technique include vacuum freeze-drying, infrared-radiation drying, spray drying, and combined drying (infrared combined with hot-air drying, etc.) [36,37,38]. Tian et al. [39] investigated the effects of different drying temperatures on the water content and total flavonoid content of sea buckthorn marc under hot-air drying conditions. The results revealed that, when the drying temperature increased, the water content and total flavonoid content of sea buckthorn marc decreased. Consequently, in order to preserve the nutrients in sea buckthorn, it is important to take the drying rate into account [40]. The drying kinetics and nutritional value of sea buckthorn were researched by Araya-Farias et al. [41] at different hot-air drying and vacuum freeze-drying temperatures. They discovered that the lower drying temperature, the better the nutrient retention. Meanwhile, vacuum freeze-drying is more effective than hot-air drying at preserving the nutrients in sea buckthorn. However, the substantial energy expenditure of vacuum freeze-drying limits its utilization in the sea buckthorn drying industry [42]. For the sake of solving the issue of energy costs. Geng et al. [43] studied the drying kinetics, physicochemical characteristics, and microstructure of sea buckthorn using hot-air drying, infrared-radiation drying, combined infrared and hot-air drying, pulsed vacuum-drying, and vacuum freeze-drying methods. The results indicated that pulsed vacuum-drying retained slightly fewer nutrients in sea buckthorn than vacuum freeze-drying. However, it is rather inexpensive and has the potential to be unscaled for industrial production.

The surface of sea buckthorn is covered with a waxy layer [44], preventing internal moisture from migrating to the surface during drying. The drying process will, therefore, take longer and use more energy to remove the water. There is also a risk that the drying quality will worsen as the drying duration increases [45]. It is, therefore, necessary to employ pretreatment techniques to reduce the hydrophobicity of the sea buckthorn epidermis before drying begins. Enhance the diffusion of moisture during drying [46] to speed up drying and ensure drying quality. Blanching pretreatment, ultrasonic pretreatment, mechanical pretreatment, and chemical pretreatment are now prevalent pretreatment techniques for berries before drying [47,48,49]. Yao et al. [50] investigated the comparative analysis of blanching pretreatment, ultrasonic pretreatment, and chemical pretreatment to improve the effectiveness of hot-air drying of sea buckthorn with large pulp. It was discovered that different pretreatment procedures all accelerate the drying rate by damaging the surface microstructure of the sea buckthorn. The flavonoids were damaged due to the high temperature involved in the blanching pretreatment, resulting in a substantially lower content compared with other preprocessing methods. Ni et al. [51] researched the effects of ultrasonic pretreatment and chemical pretreatment on the drying characteristics and microstructure of goji berry under electro-hydrodynamic drying (EHD) conditions. The outcomes showed that each pretreatment method combined with EHD drying was able to accelerate the drying speed of goji. It maintains good drying quality while saving energy. However, the surface of chemical pretreatment may be contaminated with chemical residual reagents, posing food-safety concerns [52].

Sea buckthorn has been the subject of much research on novel drying and pretreatment processes all around the world as a significant natural resource. This has boosted the sea buckthorn industry. These technological studies have demonstrated great potential for applications in drying agricultural products. However, several challenges still exist in the drying industry of sea buckthorn. Bugbears to date include complex drying systems, expensive equipment, demanding technical operations, etc. The promotion and use of innovative drying and pretreatment methods have been hampered by the aforementioned problems. Therefore, how to strike a balance between economic cost and quality is a critical problem to consider during the sea buckthorn drying process. Hence, the aims of this study are outlined below:

(1) Provide an overview and analysis of different drying and pretreatment techniques for sea buckthorn. The drying dynamics, physical and chemical characteristics, microstructure, and nutrient content of sea buckthorn are assessed using single drying, combined drying, and pretreatment techniques. We will analyse the effects of different drying and pretreatment techniques on drying characteristic and quality.
(2) Summarise the current problems in the field of sea buckthorn drying.
(3) Analyse the current situation of superior quality processing of sea buckthorn products and the way to achieve it.
(4) Summarise and forecast the main trends in sea buckthorn drying, pretreatment procedures, and high-quality processing.

## 2. Research Progress of Sea Buckthorn Pretreatment Technology

**Figure 1:**
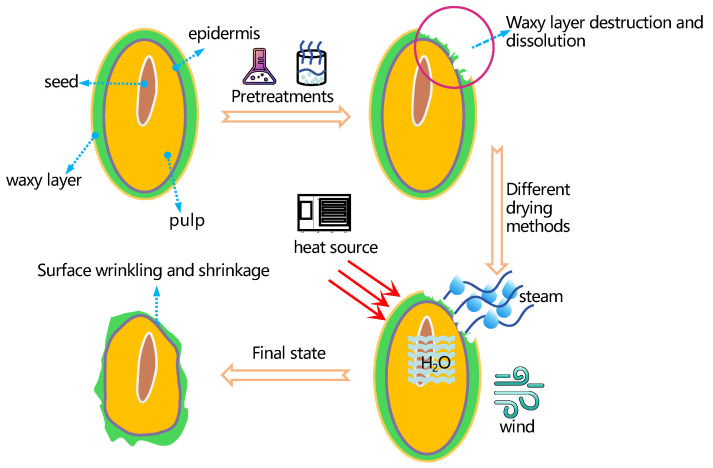
Changes in the surface of sea buckthorn pulp after pretreatment and drying.

There has been a lot of research conducted on the biological components found in sea buckthorn berries. However, the microstructure of sea buckthorn has not been well covered. The cuticle is the outermost covering of the plant’s surface. It is made up of both the outer and interior epidermal waxes [53]. The presence of a waxy layer is essential for berry plants. It minimises water evaporation from the plant’s core and protects the plant against elements such as radiation and insect infestations [54]. In reality, however, the waxy layer must be removed to accelerate drying [55]. The structure of a sea buckthorn pulp profile is depicted in Figure 1. The changes in the surface structure and wax-layer structure of sea buckthorn pulp following pretreatment and drying are expressed. The breakdown of the surface structure by physical and chemical means is referred to as pretreatment before drying, so as to achieve the goal of increasing the drying rate, improving the colour of the food, reducing nutrient loss, and so on. The effects of eight different pretreatment methods, such as blanching, ultrasonic, prick holes, and alkaline solution on the drying characteristics and quality of sea buckthorn are shown in Table 1 below.

### 2.1. Chemical Pretreatment

Depending on the physical state, chemical pretreatment can be divided into liquid and gas phases. Examples include hypertonic solution, alkaline solution, acidic solution, ozone, etc. [56,57,58,59]. The alkaline solution of the chemical pretreatment of sea buckthorn before drying has received the most attention in research. These alkaline solutions comprise 2% Na_2_CO_3_, 2% NaHCO_3_, 2% drier, etc. It not only damages the microstructure of the surface waxy layer but also partially disrupts intracellular binding via pectin denaturation [60,61]. This is why it has the ability to accelerate the drying process while reducing the possibility of quality damage. Yao et al. [50] discovered that 2% Na_2_CO_3_ resulted in the most significant disintegration of the waxy layer and degradation of the stomatal structure on the fruit’s surface. With a 62.71% shorter drying time than the control, this pretreatment had the quickest drying time of the others. This conclusion is comparable to that of Song et al. [62] for goji drying and pretreatment. The total phenolic content increased by 6% and the total flavonoid content by 35.7% when compared to the control. The drying rates and drying quality for the remaining alkaline solution pretreatments are listed in detail in Table 1 below.

The key elements influencing alkaline solution pretreatment include solution concentration, pH value, pretreatment time, temperature, solution type, etc. The concentration of the alkaline solution can influence the organisation of the berries as well as their nutrient content. Higher solution concentrations can more effectively modify the microstructure of berries, resulting in a faster drying rate. Different pretreatment times may be required for different materials and drying effects. However, as the pretreatment period increases, the greater the degree of microstructural changes on the material surface, the more severe the tissue collapse. This is in agreement with the findings of the drying study of pumpkin flakes by Çağlayan et al. [63]. On the other hand, raising the temperature of the chemical pretreatment will degrade the quality of the dried products. This is consistent with the findings of the Pahlavanzadeh et al. [64] investigation. Therefore, elements such as chemical pretreatment time, solution concentration, and temperature should be chosen based on the unique material type and target product requirements. The aforementioned characteristics should be considered when choosing the optimal pretreatment scheme.

Another chemical pretreatment currently used for berries is acid solution pretreatment. The principle is to enhance drying quality through the activity of passivated enzymes. Pirone et al. [65] investigated the effects of several pretreatments on the drying characteristics and colour of cherries. These pretreatment methods include blanching (exposure to water vapour at 100 °C during 1.5 min and subsequent immersion in cool water at 4 °C for 2 min), blanching + acid solution (soak in a citric acid solution (10% *w*/*v*) for 5 min after blanching), and blanching + mixed solution (soak in a citric acid solution (10% *w*/*v*) and calcium lactate (2.5% *w*/*v*) for 5 min after blanching). Results indicated that blanching + acidic solution and blanching + mixed solution immersion could shorten drying time under hot-air drying conditions of 70 °C, 4 m/s wind speed, and 8% relative humidity. It outperformed the control group in terms of colour retention. In years to come, sea buckthorn could attempt pretreatment with an acidic solution to discover its effects on drying properties and quality.

However, chemical pretreatment has the same limitations. This may have an impact on the nutrient content. Vásquez-Parra et al. [66] investigated the influence of chemical and physical pretreatments on currant convection drying. When compared to fresh samples, pretreatment with 1.5% NaOH solution + 4.74% olive oil considerably reduces the vitamin C content of currants by 0.14 (mg/g db). Meanwhile, after processing, its epidermis may contain chemical residues that constitute a food safety issue.

### 2.2. Physical Pretreatment

Physical pretreatment is mainly divided into thermal and non-thermal treatments. They include microwave, blanching, ultrasonic, steam blasting, and pulsed electric field [67,68,69]. Physical pretreatment methods for sea buckthorn currently include blanching, piercing, ultrasonic waves, etc. The principle of action is the same as that of chemical pretreatment. All of them are to destroy the waxy layer and microstructure of the sea buckthorn surface, allowing moisture to readily reach the surface and hasten drying. New pretreatment technology has had tremendous development potential in recent years. High-humidity hot-air blanching is part of the novel thermal pretreatment [70]. Non-thermal pretreatment methods include pulsed electric fields, among others. The detailed parameters and conclusions of the specific physical pretreatment are listed in Table 1 below.

To evaluate the influence of physical pretreatment on sea buckthorn drying. Araya-Farias et al. [71] explored the promotion of osmotic dehydration of sea buckthorn pulp via pretreatment. They discovered that steam-scalding pretreatment increased the rate of osmotic dehydration by raising the sugar content. Under hot-air drying conditions, Yao et al. [50] researched the effects of blanching and ultrasonic pretreatment methods on the drying characteristics and quality of sea buckthorn with large pulp. The results revealed that the 70 °C blanching pretreatment damaged the microstructure of the waxy layer on the surface of the sea buckthorn and increased the drying rate significantly. The 150 W ultrasonic pretreatment extended the drying time rather than accelerating the drying rate. This could be because the ultrasonic time is too brief, causing the cells to become swollen and insufficient to form microchannels. It hinders water migration and decreases the rate of drying.

Pretreatment time, temperature, and power are all factors that influence physical pretreatment. Under certain conditions, the drying rate will be faster with longer physical pretreatment and higher temperatures. However, when compared to the low temperature and control groups, the colour difference value will grow. Meanwhile, rising temperatures promote water oscillation and the rupture of microbubbles. It causes an increase in the cell gap and accelerates the release of flavonoid. This is in agreement with the findings of Song et al. [72]. The higher the microwave power, the shorter the time required to reach the same moisture content and the faster the drying. This is consistent with the findings of Horuz et al. [73].

Other physical pretreatment techniques used on berries recently include pulsed electric field (PEF), osmotic dehydration (OD), and others [29]. Prothon et al. [74] studied the effect of osmotic pretreatment (sucrose) on the drying characteristics and quality of apples under microwaves and hot-air drying. They came to the conclusion that the osmotic pretreatment shortened the drying time. The product has improved in terms of hardness, crispness, and rehydration. Yu et al. [75] evaluated the effect of PEF pretreatment on blueberry drying properties and nutrient content under hot-air drying (45 °C, 60 °C, and 75 °C) and vacuum-drying conditions. The results revealed that PEF pretreatment had no discernible impact on the nutrient content and could reduce the drying time of blueberries. The new physical pretreatment procedures described above have the potential to be used in the drying pretreatment of sea buckthorn.

However, the physical pretreatment methods also have limitations. As previously stated, strong ultrasonic power causes cellular tissue to expand, making water escape difficult. This prolongs the drying time and reduces the rehydration rate [76,77]. Meanwhile, hot-water blanching produces excessive waste-water and causes nutritional losses. The findings are consistent with those of Wang et al. [78].

### 2.3. Combined Pretreatment

The combination of two or more single pretreatment techniques is referred to as combined pretreatment. The common combined pretreatment methods for fruits and vegetables are chemical + ultrasonic pretreatment, chemical + prick holes pretreatment, chemical + physical pretreatment, and ultrasonic + ultra-high pressure [79,80,81].

Fewer investigations have been conducted on the combined pretreatment of sea buckthorn before drying. Tan et al. [81] researched the effect of single pretreatment versus combined pretreatment methods on the drying characteristics and quality of sea buckthorn under 60 °C and 2.2 m/s wind speed hot-air-drying conditions. The single pretreatment method is to prick different numbers of holes. The combined pretreatment methods are as follows: ➀ 3% Na_2_CO_3_ + 40 kHz, 200 W ultrasonic; ➁ 3% Na_2_CO_3_ + 40 kHz, 200 W ultrasonic + prick holes. The results suggested that the prick-holes pretreatment resulted in mechanical injury to the epidermis. Although the drying time was reduced by 2.33% to 21.82% compared to the control group, this will lead to a decrease in the sensory score values of sea buckthorn pulp after drying. The Δ*E* value decreases and subsequently rises as the number of prick holes increases. The pretreatment using the prick-three-holes method has the lowest Δ*E* value. This may be related to the shorter drying time and the fewer browning reactions. The surface waxy layer gradually melts and micropores emerge after increasing the ultrasonic treatment period from five to fifteen minutes with the combined drying group ➀. It cuts drying time by 5.08% to 6.14% when compared to the control group, suggesting that ultrasonic time has no significant impact on drying rate. However, with the ultrasonic time enhanced, the total flavonoid content increased by 6.88% and 14.56%, respectively. Therefore, the combined pretreatment of ultrasonic + alkaline solution is beneficial to increase the total flavonoid content. In the combined pretreatment group ➁, when the ultrasonic time was extended to 15 min, the destruction of the surface stomata and only the skeleton were visible. The drying time was reduced by 30.30% to 33.05% compared to the control group. After the ultrasonic time was increased to 15 min and the number of prick holes reached six, the sea buckthorn sample had the highest L* and B* values and the lowest ∆*E* values. This phenomenon demonstrates that the combined pretreatment group ➀ could cause the release of phenolics. Meanwhile, accelerating the drying speed using pricking-holes pretreatment could reduce the loss of phenolics. The detailed drying characteristics are analysed as illustrated in Table 1 below.

It is evident that the most effective pretreatment combination could be chosen by conducting experiments to confirm the drying qualities and characteristics under various pretreatment parameter combinations. In this way, we can speed up the drying rate while at the same time ensuring good drying quality. This is in line with the results of Santos et al. [82] for carrots in the combined pretreatment study. The study of combined pretreatment of sea buckthorn drying will reduce production costs while increasing product value while accelerating drying rate and maintaining better drying quality than single drying.

**Table 1:** Effects of different pretreatment methods on drying characteristics and quality of sea buckthorn.

Pretreatment Methods	Type	Drying Characteristics and Quality	Reference
70 °C blanching pretreatment	Physical pretreatment	The waxy layer of sea buckthorn epidermis was partially dissolved and showed few breaks and cracks, and the drying rate increased significantly. However, the total flavonoid content was slightly lower than that of the control, and the total phenolics content decreased by about 1.4%.	[50]
Prick hole pretreatment	Physical pretreatment	The drying time was reduced by about 3.4%, 18%, and 24.4% when the number of prick holes was 1, 3, and 6, respectively, compared to the control group. And, it will have a higher rehydration rate and a smaller colour difference value Δ*E* than the colour closest to fresh sea buckthorn.	[83]
2% drier	Chemical pretreatment	The waxy layer on the surface of the sea buckthorn had disappeared in dissolution, and there were slight cracks near the stomata. The water content was 22.98% higher than the control in the first 36 h of drying. In comparison to fresh samples, the total flavonoid content increased by 35.7%, the total phenolics content increased by 2%, and the brightness value L* decreased by 38.7%. The colour difference value Δ*E* added to 9.37% in comparison to the control.	[50]
2% Na_2_CO_3_	Chemical pretreatment	The surface waxy layer structure of sea buckthorn has been severely damaged, resulting in dissolution and a large number of cracks. The drying rate was significantly increased. Compared with the control group, the drying time was reduced by 62.17%, the browning index was decreased by 54.5%, the total flavonoid content was increased by 35.7%, and the total phenolics content was enhanced by 6% but was lower than that of the fresh samples.	[50]
2% NaHCO_3_	Chemical pretreatment	The dissolution of the waxy layer in the epidermis of sea buckthorn was relatively slight, and stomatal disruption was small. The drying rate significantly increased. At 36 h before drying, the moisture content was reduced by 18.53% more than the control. In comparison to the control group, the colour difference value Δ*E* was lowered by 13.4% and the L* value raised by 4.6%. The total flavonoid level was marginally greater than in fresh samples, and the total phenolic content was 5.4% higher than in controls.	[50]
150 W Ultrasonic	Physical pretreatment	There was dissolution of the waxy layer of the sea buckthorn epidermis, but no breaks or cracks were present. The drying rate was lower than in the control group. At 36 h before drying, 1.29% less water content was removed, and the total colour difference value Δ*E* was reduced by 2.8% compared to the control. The browning index and ascorbic acid content were slightly lower than the control, and the differences were not significant. The total flavonoid content increased by 25%, and the total phenol content was elevated by 2.7%.	[84]
150 W Ultrasonic + 3% Na_2_CO_3_	Combined pretreatment	The waxy layer on the surface gradually dissolved, and micropores appeared at 15 min. It has accelerated the drying rate by 1–2 min compared to the control group. The total colour difference value Δ*E* decreased by 13–5.7% as the ultrasonic time increased from 5 to 15 min compared to the control group. The content of total phenolics was highest at 5 min. The total phenolics content gradually decreased with time and was lower than the control at 15 min.	[81]
150 W Ultrasonic + 3% Na_2_CO_3_ + Prick hole	Combined pretreatment	The surface stomata of sea buckthorn were destroyed, and only the skeleton was visible. It drastically reduced drying time. When compared to the control, the greatest drying rate can be enhanced by up to 37.5% for different duration combinations. The total colour difference value Δ*E* can be reduced by up to 22.9%, and the total flavonoid content can be increased by about 6.1–14.8%.	[81]

As analysed in Table 1, all the above pretreatment methods destroyed the waxy layer of sea buckthorn. It is convenient for the internal moisture to reach the surface quickly and then evaporate, so as to speed up the drying rate. The researchers used blanching to remove the waxy layer, but blanching will cause too much nutrient loss due to the extreme surface temperature. Excessive water usage and effluent discharge are also troublesome issues if used in the drying industry. Chemical pretreatment and physical pretreatment are compared with convenience, economics, and other advantages. However, during pretreatment of the material, the surface will be left with chemical reagents, posing a threat to food safety. The later wastewater treatment problems will also pose a threat to the environment, just like the blanching pretreatment [85]. In recent years, researchers have begun to apply combined pretreatments to sea buckthorn drying. Their impact on the characteristics and quality of sea buckthorn drying has been investigated to find a balance between economics, ecology, and efficiency. The differences between laboratory and actual production should be recognised for the pretreatment technique before sea-buckthorn drying. The effects of new pretreatment technologies such as high-pressure processing (HPP), pulsed electric field (PEF), and acidic solution on the drying characteristics and quality of sea buckthorn should be studied. Combining the different advantages of physical and chemical pretreatment, the optimal pretreatment of sea buckthorn under different drying techniques should be sought. In the future, research should focus on the application of joint pretreatment techniques. This allows us to strike a balance and apply it to large-scale industrialised manufacturing, boosting the quality and efficiency of sea buckthorn drying.

## 3. Research Progress of Sea Buckthorn Drying Technology

**Figure 2:**
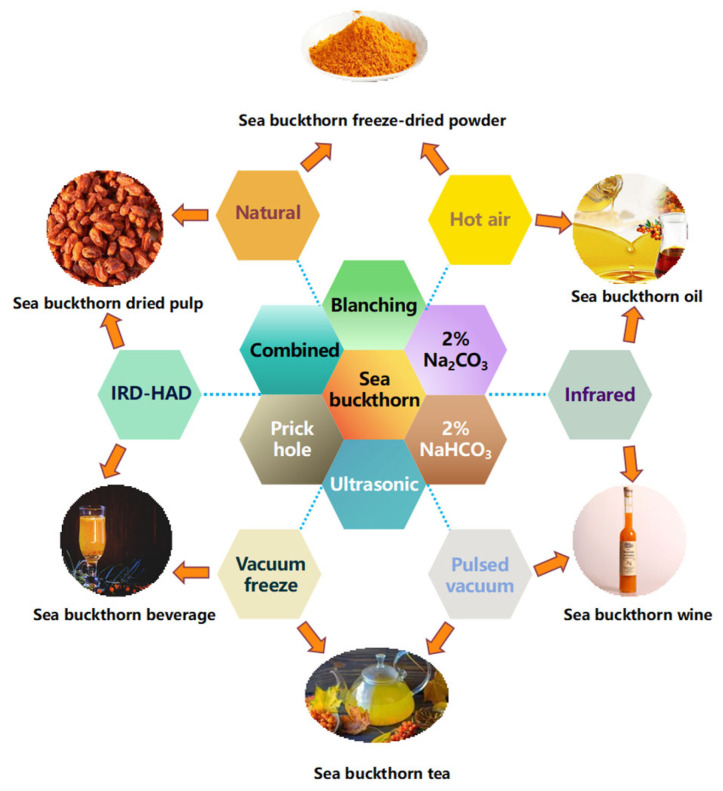
Application examples of sea buckthorn high-quality processing and products.

The evaporation of water within the material through the migration path to the outside is the essence of the drying process [86]. Examples of the application and implementation of high-quality processing of sea buckthorn products are shown in Figure 2. In this section, the different drying techniques for sea buckthorn are first introduced. Secondly, its drying characteristics are studied. The drying characteristic curve is used to study the comparative analysis of drying rates under different drying procedures. The energy consumption of different drying techniques is investigated using energy consumption measurements. The destruction of cell tissues and the establishment of water diffusion channels using different drying techniques are studied through microstructure changes. The effects of different drying techniques on the quality of sea buckthorn are investigated through changes in nutrient content, colour, and lustre [87]. Finally, the trends in the development of different drying techniques for sea buckthorn are analysed to provide ideas for the establishment of relevant drying models for sea buckthorn. Table 2 compares the effects of several drying processes on the drying characteristics and quality of sea buckthorn.

### 3.1. Natural Drying

Natural drying is a traditional drying process [88] that is classified into two types: natural sun-drying and natural shadow-drying [89]. Natural sun-drying involves placing the material flat in a material tray or in a large field. It evaporates material moisture by utilising sun radiation and air flow. Natural shade-drying is putting the material in a cool and ventilated place. Drying is achieved via the evaporation of water through air flow [90]. Natural drying mostly utilises solar and wind energy, which are renewable, low-cost, and non-polluting [91,92,93].

Natural drying is a traditional and widely used drying technique. Current technological studies of natural sea buckthorn drying have concentrated on drying times and quality. Li et al. [94] studied the effects of natural drying, hot-air drying, and vacuum freeze-drying on the flavonoids and phenolics of various Chinese sea buckthorn types. According to the findings, the TPC (total phenolics content) content after natural drying was lower than that after hot-air drying and vacuum freeze-drying. This is due to the fact that the detrimental effect of temperature on sea buckthorn phenolics is weaker than that of drying time. The difference in TFC (total flavonoids content) content was not significant, indicating that the drying process has little effect on total flavonoid content. Lin et al. [95] researched the effects of natural drying, hot-air drying, heat-pump drying, and vacuum freeze-drying on the drying characteristics and quality of sea buckthorn pulp. The effects of different drying methods on the drying properties and quality of sea buckthorn pulp are reflected in the drying characteristic curves, colour, browning index, and nutrient content. They discovered that prolonged exposure to air during natural shade drying and sun drying resulted in severe colour degradation and an inadequate nutrient retention rate. Of these, natural shade drying has the lowest drying rate. Natural sun-drying sea buckthorn pulp has higher TFC levels than fresh pulp and other drying processes but has the highest browning index.

The main disadvantages of natural drying are long exposure to air and vulnerability to weather and other factors. With limited drying efficiency, it cannot guarantee drying quality [96,97]. It is clear that it does not match the requirements of modern agricultural growth.

### 3.2. Hot-Air Drying

Hot-air drying is one of the most widely used drying techniques in the drying industry today. It works on the basis that the air is heated by a heat source, and the hot air is transferred to the surface of the material through thermal convection. The surface moisture is evaporated to achieve the drying purpose [98]. The two most important parameters influencing the drying rate of hot air are air temperature and velocity. Between them, temperature is the main influencing factor [99]. Hot-air drying is now widely used in the drying industry [100]. It has the advantages of low cost, mature technology, and simple operation [101].

Most of the current research on hot-air drying of sea buckthorn focuses on quality optimisation of the dried product. The optimal drying process of hot-air drying technology is explored through the study of its sensory, microstructure, and nutrient content. In order to explore the effects of different temperatures on the drying characteristics and quality of sea buckthorn under hot-air drying conditions, Stoica-Guzun et al. [102] researched the effects of different pretreatment processes on the drying characteristics of sea buckthorn pomace, the extraction of sea buckthorn seed oil, and the composition of the oil under the hot-air drying conditions (40 °C, 50 °C). The results suggested that the effective diffusion coefficient increased with the thickness of the slag layer and the drying and pretreatment temperatures. The drying temperature of the hot air had no effect on the average drying rate. Based on the former two different drying temperatures, Guan et al. [103] studied the effects of four different drying temperatures (50 °C, 60 °C, 80 °C, and 100 °C) under hot-air drying conditions on the total phenolics, total carotenoids, and total chlorophyll levels in sea buckthorn leaves. The results indicated that the drying time was reduced from 250 min to 60 min with the increase in drying temperature. For the sake of discovering the difference between the hot-air drying method and other drying methods, Kyriakopoulou et al. [104] researched the impact of three different drying methods (hot-air drying at 50 °C ± 2 °C and 1 m/s; vacuum freeze-drying at −30 °C; and accelerated solar-drying) on the quality and antioxidant activity of sea buckthorn pulp and leaves. The data revealed that the residual moisture content of sea buckthorn after hot-air drying was considerable due to the presence of floating saturated air in the hot-air dryer. By analysing the drying kinetic curves of sea buckthorn pulp, it can be concluded that hot-air drying is the most rapid and efficient of the three methods, but it has a greater impact on the colour and microstructure of sea buckthorn.

As a result of prolonged exposure to high temperatures and oxygenated conditions, hot-air drying causes serious nutrient loss in the sea buckthorn pulp and more severe colour and structural damage. However, owing to its advantages, it is still the market’s mainstream at the moment. Hot-air drying is a great advance in drying techniques compared to natural drying. Consequently, further exploration and innovation are still needed to improve the drying technique.

### 3.3. Heat-Pump Drying

It is a well-established fact that heat can only be transferred automatically from a source at a higher temperature to a source at a lower temperature, but not in the opposite direction. Nonetheless, a heat pump is a mechanism that consumes some of its own energy to assimilate heat from a low-temperature heat source and then releases heat, which can be put to use at a higher temperature. Since the heat obtained exceeds the energy consumed, heat-pump drying is an energy-conserving device. In comparison with traditional drying techniques, the method offers benefits such as higher energy efficiency, reduced operational expenses, superior product quality, and the absence of environmental pollution [105,106].

Heat pump drying, a new technology that improves on hot-air drying, is barely studied in sea buckthorn nowadays. To assess the impacts of heat-pump drying on the drying properties and quality of sea buckthorn pulp, Lin et al. [95] evaluated the impacts of five different drying methods on the drying properties and quality of sea buckthorn pulp containing larger fruits. The findings illustrated that the drying rate of the heat pump was comparable to, but marginally slower than, that of the hot air. Heat-pump drying resulted in a reduced colour difference value Δ*E*, lower browning index, and increased V_C_ content when compared to hot-air drying. The total phenolics and total flavonoid contents were notably higher than that of hot-air drying and natural drying. However, further studies are needed to understand these outcomes.

Regarding current heat-pump drying technology, it is primarily low-temperature drying. Consequently, it takes longer to dry compared to high-temperature drying, and the equipment investment is relatively costly. We ought to research the energy consumption and drying process in an attempt to advance research on sea buckthorn drying. As an enhancement over hot-air drying, it has tremendous practical production potential.

### 3.4. Infrared-Radiation Drying

Infrared-radiation drying is a modern, energy-efficient, and ecologically beneficial drying process. It provides the advantages of even heating, powerful penetration ability, a high heat-transfer rate, and a swift drying rate [107,108]. The rationale for drying is that radiation contacts the material’s surface and resonates with inorganic and organic macromolecules. After resonance, these molecules are accelerated and brush against one another, generating heat, and promoting material drying [109].

On the one hand, studies on the drying of sea buckthorn using infrared radiation techniques have focused on exploring the variation in energy consumption with different working conditions. On the other hand, the concentration is on the search for the optimal drying process. Geng et al. [43] investigated the influence of different drying methods on the drying kinetics, physicochemical properties, and microstructure of sea buckthorn. The outcome was that infrared-radiation (IR) drying (300 W, 60 °C) had a faster drying rate in the early stages than hot-air drying (60 °C, 2.2 m/s), but the entire drying time was longer. This is most likely due to the strong penetration power of infrared radiation, which causes rapid heat generation inside the material. However, this destroys the internal structure of the sea buckthorn and reduces the rate of rehydration. This is consistent with the results of Zhang et al. [77]. By analysing the microstructure of the epidermis, the epidermis after IR drying is smooth but has some holes. The same phenomenon was observed by Huang et al. [110] in their study of the impact of far-infrared-radiation drying on the drying characteristics and quality of stevia. Infrared-radiation drying maintained the total flavonoid, total phenolics, and ascorbic acid content better than hot-air drying and infrared assisted hot-air drying. It has a smaller colour difference value Δ*E*, browning index, and a higher brightness L*. The above index values, however, are inferior to those of vacuum freeze-drying and pulsed vacuum drying.

It is evident that infrared radiation drying is not applicable to the pre-drying stage of sea buckthorn, although it has the advantages of a rapid drying rate, better retention of nutrients and colour, and low energy consumption [111]. This is because of its strong penetrating power, which easily causes rupture and thermal damage to sea buckthorn, and excessive heating phenomena. In future applications of infrared drying techniques to sea buckthorn drying, different drying techniques can be combined to achieve better drying results by combining their respective strengths.

### 3.5. Spray Drying

The spray-drying method is based on an atomiser, which changes the material’s liquid condition to a spray state (fine dispersion state). The enlarged surface area allows the moisture in the droplet to be instantly evaporated by the hot air flow during settling, thus completing the drying process. Compared with the liquid state, the powder state is more stable and has more advantages in storage [112,113]. Moreover, it has the advantages of a quick drying rate and simple process [114].

Owing to the peculiar features of spray drying, the current study on spray drying of sea buckthorn focuses mostly on converting sea buckthorn beverages into sea buckthorn powder or making sea buckthorn oil microencapsulated. Selvamuthukumaran et al. [115] used the response surface methodology to optimise the spray-drying process of sea buckthorn pulp beverages. Moisture content, solubility, dispersion, vitamin C content, and overall colour difference values were used as five response surface models. The optimal spray-drying process was obtained by superimposing the contour plots: an inlet air temperature of 162.5 °C and 25 g of maltodextrin per 100 g of beverage. Čulina et al. [116] optimised the spray drying microencapsulation process of sea buckthorn pulp oil in their study. Increasing the inlet temperature during spray drying boosted product yield, encapsulation efficiency, and solubility, according to their studies. However, it decreased the moisture content, total carotenoids, and antioxidant capacity of the powder. It was found that the best conditions for the production of sea buckthorn pulp oil powder were when the inlet temperature was 120 °C, β-cyclodextrin was used as the carrier, and the ratio of carrier to sea buckthorn pulp oil was 4.

Spray drying can dry liquid materials into a solid powder that is suitable for storage. The downsides, however, are also obvious. The inlet drying temperature is high (150–220 °C), which can damage nutrients such as vitamin C [114]. Furthermore, the energy consumption is high, the equipment is large, and it is prone to problems such as sticking to the inner wall.

### 3.6. Pulsed Vacuum Drying

Vacuum drying is a drying technique that involves placing materials under negative pressure and heating them to reach the boiling point under a negative pressure state, or cooling them to solidify the materials, and then drying them. As the material is located in a closed vacuum environment, it can effectively isolate the air to avoid oxidation reactions. Ensuring better drying quality of the material than traditional drying methods [117,118]. Pulsed vacuum drying functions on the basis of vacuum drying, through the control of vacuum and atmospheric pressure environment pulsed cycle, so that the material inside the microscopic pores are constantly squeezed and expanded. As a result, micro-channels are formed. It can significantly improve the moisture migration rate.

As the air is isolated under negative pressure, the material is better able to maintain its original character. Therefore, research on vacuum-drying techniques for sea buckthorn pulp has mainly focused on energy consumption and drying quality. Araya-Farias et al. [71] studied the drying kinetics and nutrient content of sea buckthorn pulp under the conditions of vacuum drying (50 °C, 25 in Hg) and hot-air drying (1 m/s, 50 °C) following liquid nitrogen pretreatment and osmotic dehydration with sucrose solution. The results indicated that vacuum drying was approximately 43% slower and reduced total carotene by 0.24 mg/100 g compared to hot-air drying but increased total phenolics and V_C_ by 24.4 mg/100 g and 2.24 mg/100 g, respectively. On the basis of vacuum drying. Geng et al. [43] investigated the effects of hot-air drying, infrared drying, infrared-assisted hot-air drying, pulsed vacuum drying, and vacuum freeze-drying on the drying kinetics, physicochemical properties, and microstructure of sea buckthorn. They found that both pulsed vacuum drying and vacuum freeze-drying could maintain high ascorbic acid and total phenol contents, as well as superior rehydration capacity and colour. However, pulsed vacuum drying was more cost-effective in comparison and has more potential for industrial applications.

Pulsed vacuum-drying techniques are normally combined with microwave or infrared drying and other drying methods because of the slow drying rate. To improve drying efficiency and acquire a preferred drying quality, the drying time should be shortened to improve drying efficiency. Future developments in vacuum drying, similar to infrared drying, need to be applied in combination with other drying techniques to seek the optimal drying process.

### 3.7. Vacuum Freeze Drying

Vacuum freeze drying has been a modern technology applied to sea buckthorn drying in recent years. The drying process is divided into three stages based on the three-state shift in water [119,120]. The first stage is pre-freezing. That is, freezing the material below the eutectic point temperature transforms the free water inside the material from a liquid state to solid state. The second stage is the sublimation drying stage. The frozen free water inside the material is converted to the gaseous state via sublimation under a vacuum [121]. The third stage is the resolution drying stage. The bound water in the material is removed via resolution drying, so that the water completes the migration and then completes the drying process.

Vacuum freeze-drying technique is effective in maintaining the quality of products and nutrients after drying. Presently, research on vacuum freeze drying of sea buckthorn has evolved from drying rates, quality, and energy consumption to nutrient and active-component extraction. Li et al. [94] studied the effects of natural drying, hot-air drying, and vacuum freeze-drying on the phenolics content and antioxidant activity of different varieties of sea buckthorn in China. The data suggested that vacuum freeze drying had a 1.56–2.97-times-higher total phenolics content than the other drying processes. Furthermore, overall flavonoid retention was superior to the other drying procedures. In an attempt to determine the impact of drying methods on the extraction rate and quality of sea buckthorn oil from sea buckthorn seeds and fruits in Quebec, Gutierrez et al. [33] conducted 24 h of vacuum freeze-drying, utilizing both hot air drying (50 °C, 1.0 m/s) and vacuum freeze drying (using a vacuum pressure of 0.0145 kPa at a constant temperature of 50 °C in the freeze dryer and a condenser temperature of −48 °C). The results of the study indicated that the extraction rate of sea buckthorn seed oil was similar after hot-air drying and vacuum freeze-drying. But, there was a significant difference in the extraction rate of sea buckthorn pulp oil. The vacuum freeze-drying rate was 23.8% lower than the hot-air drying extraction rate. Notably, the oil extracted from sea buckthorn pulp after vacuum freeze-drying had a lower peroxide value. This phenomenon demonstrates that vacuum freeze drying enhances the quality of sea buckthorn oil.

The vacuum freeze-drying technique consumes a lot of energy and has a long production cycle [122]. This is due to the fact that there is no heat convection in a vacuum environment, and heat and mass transfer are extremely slow, causing energy consumption and costs to rise and drying rates to fall. From the point of view of industrial large-scale production, the long drying time leading to high costs is the major disadvantage of the sea buckthorn vacuum freeze-drying technique. In the future, this problem can be optimized via pretreatment or intelligent, new online-measurement and control techniques [123]. Accelerated drying rates by finding the optimal pretreatment process under vacuum freeze-drying conditions. Through the intelligent, new online-measurement and control technology, we can grasp the moisture migration situation and make an accurate judgement of the drying end point.

### 3.8. Combined Drying

**Figure 3:**
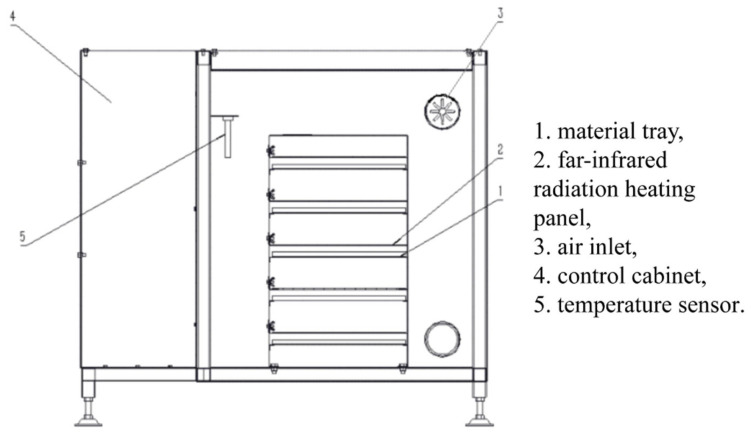
The two-dimensional structure of the infrared-radiation-assisted hot-air dryer [43].

Combined drying is a drying technique that combines two or more single drying methods. Under the premise of ensuring the quality of sea buckthorn drying, the advantages complement each other, shorten the drying time and save the cost. Until now, the only combined drying technique that has been applied to sea buckthorn is infrared-assisted hot-air drying. The two-dimensional structure of the infrared-radiation-assisted hot-air dryer is shown in Figure 3 [43].

Infrared-assisted hot-air drying is a combined drying method with quick heating efficiency and energy savings. The approach effectively enhances the drying rate and product quality of agricultural produce and foodstuffs, specifically in the case of sea buckthorn. At the moment, infrared-assisted hot-air drying for sea buckthorn is mostly focused on drying rate and quality research, as well as the development of drying equipment. Geng et al. [43] investigated the effects of hot air drying, infrared drying, infrared-assisted hot-air drying, pulsed vacuum drying, and vacuum freeze-drying on the drying kinetics, physicochemical properties, and microstructure of sea buckthorn. The obtained experimental data revealed that, among the above drying techniques, infrared-assisted hot-air drying had the shortest time to complete the drying process. This is because infrared radiation is able to penetrate the surface of the material and remove moisture from sea buckthorn that is difficult to remove via heat conduction. Microcracks and pores are observed on the surface of sea buckthorn after infrared-assisted hot-air drying. As a result, the total phenolics content, total flavonoid content, and rehydration of sea buckthorn were higher than that of hot-air drying. This is proof that it improves the quality of drying. This aligns with Vishwanathan et al.’s research [124], which found that the use of infrared-assisted hot-air drying reduced drying time by approximately 48% when compared to single hot air drying. However, the energy consumption of infrared-assisted hot-air drying technique used in sea buckthorn requires further exploration. Motevali et al. [111] have demonstrated that the infrared assisted hot air drying of mushroom slices consumes less energy than single drying methods such as infrared drying and hot-air drying.

Although infrared-assisted hot-air drying has the potential to accelerate drying rates and reduce costs, it can also lead to crusting on the material’s surface [125], thereby hindering moisture transfer and extending drying time. Other combined drying methods for berries, such as microwave-assisted hot-air drying, microwave-assisted vacuum drying, and vacuum–freeze hot-air drying are also available for sea buckthorn drying [126,127]. Combining hot-air and microwave drying can speed up the drying process and effectively preserve nutrients. To compensate for the low drying rate during the final stage of vacuum freeze-drying, vacuum–freeze hot-air drying can be used for high-quality drying. These combined drying methods demonstrate great potential for the drying of sea buckthorn. Future combined drying techniques for sea buckthorn could be centred around the above drying methods. The balance between the drying kinetics, quality, and cost is explored to find the optimal drying process.

**Table 2:** Comparison of drying and quality characteristics of different drying methods for sea buckthorn.

Different Drying Methods	Drying Characteristics and Quality	Reference
Natural drying	The drying rate was slow. Compared to hot-air drying, drying time increased 3.4 times for natural sun-drying and 8.4 times for natural shade-drying. The total colour difference value Δ*E* was reduced by about 13% and the total flavonoid content by 4–29.3% compared to hot-air drying.	[94,95]
Hot air drying	The hot-air drying rate increased with the rise in temperature. The damage to the microstructure was severe, resulting in a serious loss of nutrients. The total phenolics content decreased by 22.7–42.3%, and the total carotenoid content decreased by 42.6–64.6% compared to the control group.	[102,103,104]
Infrared radiation drying	The drying time was prolonged by about 7% compared with the hot-air drying at 60 °C and 2.2 m/s, the total flavonoid content was enhanced by 17.6%, and the total phenolics content was increased by 33%. The holes left behind could be observed on the surface of sea buckthorn pulp due to the high capacity of infrared radiation, which could directly penetrate the surface of sea buckthorn pulp.	[43]
Heat pump drying	The drying rate of heat-pump drying was slightly slower than that of hot-air drying. Compared to hot-air drying, heat-pump drying reduced the total colour difference value Δ*E* by 7.6% and the browning index by 16.7%. The increase in V_C_ content was slightly but significantly lower than vacuum freeze-drying and fresh samples. The total flavonoid content increased by about 83% and the total phenolic content decreased by about 66% compared to fresh samples.	[95]
Spray drying	Since the liquid is in the form of mist, the contact area with hot air is expanded, which increases the drying rate significantly. The temperature at the inlet can reach 150–220 °C, causing nutrients like V_C_ in sea buckthorn to be lost.	[115,116]
Pulsed vacuum drying	Its drying rate was higher than HAD (hot air drying), IRD (infrared radiation), and IR-HAD (infrared assisted hot air drying) in the initial stage, and the drying rate decreased in the later stage. The time was extended by 15.99% and 7.83% compared to hot-air drying and IR drying, respectively. The total colour difference value Δ*E* was similar to that of vacuum freeze-drying and 87.7% lower compared with hot-air drying, which could better retain the nutrients and active ingredients and reduce the loss of nutrients.	[43,71]
Vacuum freeze drying	In comparison to the other drying techniques mentioned above, vacuum freeze-drying has the longest drying duration and consumes the most energy. However, it also has the best retention of nutrients and the lowest total colour difference Δ*E* and browning index.	[33,94]
Infrared assisted hot air drying	The drying rate was obviously increased; compared with hot-air drying and infrared drying, the drying rate increased by 11.2% and 19.6%, respectively. Compared with hot-air drying, the browning index was 40.4% higher, the total flavonoid content was 6.6% higher, and the total phenolics content was 33.3% higher.	[43]

## 4. Different Methods to Achieve High-Quality Processing of Sea Buckthorn Products

The small sea buckthorn berry is a valuable resource. Its pulps can be used to make beverages, the skin has medicinal uses, and the seeds can be extracted for oil. Sea buckthorn pulp is both sweet and sour, and it is abundant in vitamin C, flavonoids, and other nutrients. Currently, there are two main areas of focus for the sea buckthorn industry: fresh food and frozen-fruit processing [128]. Sea buckthorn products include dried pulp, beverages, freeze-dried powder, wine, flavonoid, and oil. The range of product types is extensive. Therefore, achieving high-quality processing and improving product quality is a particularly crucial issue. This section provides a comprehensive analysis of the processing status of the aforementioned products, highlighting current issues in their development, proposing methods for achieving high-quality processing, and exploring future development trends.

### 4.1. Sea Buckthorn Beverages

The utilisation of fruits and vegetables for manufacturing functional foods has been widely acknowledged globally, owing to the beneficial health effects of their bioactive constituents [129]. Sea buckthorn drinks can be classified into two categories: sea buckthorn puree and sea buckthorn complex beverages. It is worth noting that sea buckthorn compound drinks are highly preferred as they offer improved taste and nutrition compared to sea buckthorn puree. Sterilisation is a crucial process when producing sea buckthorn beverages and can be categorised as thermal or non-thermal [26]. Traditional thermal sterilisation eliminates microorganisms and enzyme activity but simultaneously impairs the heat-sensitive components and nutrients present in the juice. At present, the prevailing sterilisation technique used in sea buckthorn beverage manufacturing is traditional thermal sterilisation. The non-thermal sterilisation technique can provide the same sterilising effect as thermal sterilisation, ensuring food safety while better retaining the nutritional content of the juice. Therefore, the industry’s future sterilisation technology development should concentrate on non-thermal techniques with the aim of enhancing the nutritional content of sea buckthorn beverages.

In addition to the use of freshly-squeezed fruit, a sea buckthorn beverage is also partly made by using sea buckthorn powder which is compounded and re-brewed. Therefore, the drying process mentioned in the previous section will affect its quality to a great extent. Research on a suitable drying process can not only improve product quality but also reduce production cost.

Sea buckthorn pulp is frequently blended with other ingredients to create composite beverages that improve taste due to its sour flavour. Moreover, it enhances the nutritional value. Maftei et al. [16] conducted research on the probiotic activity and sensory acceptability of sea buckthorn, soya milk, and inulin composite beverages. The findings demonstrate that sea buckthorn, soya milk, and inulin composite beverages are nutrient-dense and more flavourful, serving as a viable substitute for dairy or soya drinks.

Consequently, the future development of sea buckthorn beverages should mainly focus on sea buckthorn composite beverages. New non-thermal sterilisation technologies, including ultra-high static pressure, pulsed electric field, and oscillating magnetic field [130], should be implemented. Research should be undertaken to optimise the process conditions concerning colony count, enzymes, and biological activity in order to manufacture a safer, more delicious, and more nutritious sea buckthorn beverage. Meanwhile, the development of drying techniques in the sea buckthorn beverage industry in the future can be researched for different combinations of drying and pretreatment technique. Through the optimal drying process, the sea buckthorn can be dried and powdered to form a new type of sea buckthorn compound beverage with other fruit powders.

### 4.2. Sea Buckthorn Freeze-Dried Powder

Sea buckthorn freeze-dried powder is made by grinding sea buckthorn pulp into powder after vacuum freeze-drying, which can retain more nutrients in sea buckthorn. It can be turned into sea buckthorn pulp, sea buckthorn wine, and other products by brewing, fermentation, and compounding. It can also be combined with other materials to make new products, such as sea buckthorn milk tablets and sea buckthorn fruit peel, etc. It is expected to be an alternative to ordinary milk tablets and hawthorn rolls because of its high levels of V_C_ and flavonoid, immunity-boosting and beauty-enhancing properties, and abundant nutritional value.

The high-quality processing of sea buckthorn freeze-dried powder is mainly aimed at optimising the vacuum freeze-drying process. Since vacuum freeze-drying is costly. Therefore, determining the precision of controlling the temperature, controlling the drying endpoint, and improving the drying efficiency is a very critical issue. It can ensure that the process used can maximise the quality of sea buckthorn. In this regard, intelligent new measurement and control techniques [123] such as low-field nuclear magnetic resonance (LF-NMR) technology can be introduced. This method can monitor moisture data (content and migration) in the sea buckthorn pulp drying process, forecast the end of sea buckthorn fruit vacuum freeze-drying, and fully guarantee the excellence quality of sea buckthorn freeze-dried powder, achieving outstanding processing quality while reducing costs.

In the future, the high-quality processing of sea buckthorn freeze dried powder necessitates starting with the drying process and implementing LF-NMR technology. Following that, the high-quality sea buckthorn freeze-dried powder produced can be combined with other products to improve their nutritional content and achieve high-quality development.

### 4.3. Sea Buckthorn Alcohol

Sea buckthorn alcohol refers to two types: sea buckthorn baijiu and sea buckthorn fruit wine. Sea buckthorn baijiu can be made by soaking sea buckthorn pulp or adding freeze-dried powder. Sea buckthorn fruit wine is a low-alcohol beverage derived from sea buckthorn pulp via a number of steps including crushing, enzymolysis, clarifying, acid reduction, and fermentation [131,132]. Sea buckthorn alcohol has both its own nutrients and nutrients from fermentation, which is one of the sea buckthorn industry’s most important development directions.

The fragrance of alcohol is a crucial indication of its organoleptic properties. It is produced by the metabolic processes of several microorganisms, including yeasts and lactic acid bacteria. An essential pathway towards achieving high-quality sea buckthorn alcohol production is to comprehend the influence of these microorganisms on its fragrance [131]. Ma [133] demonstrated that the primary aroma compounds present in sea buckthorn alcohol at high concentrations were 3-methyl-1-butanol (15.38%), ethyl 2-hydroxypropionate (15.193%), and phenyl ethanol (7.365%). The alcohols that gave sea buckthorn fruit wine its flavour were higher alcohols, mostly normal by-products of the yeast during the fermentation process. Sea buckthorn alcohol is murky because it is made from newly squeezed sea buckthorn beverages. Clarification is, therefore, necessary to improve the flavour and stability of sea buckthorn alcohol [134]. Liu [131] has researched the influence of the clarification process of sea buckthorn juice on the scent profile of sea buckthorn alcohol. The findings indicated a 16.45% increase in the contents of 3-methyl-1-butanol and a 5.32% increase in ethyl caprylate in sea buckthorn alcohol that underwent clarification treatment followed by fermentation, compared to those fermented from raw juice without clarification treatment. The study also highlights that clarifying the sea buckthorn juice before fermentation was more effective in promoting the formation of the alcohol’s aroma.

Apart from fragrance, colour and nutrients serve as crucial physicochemical indicators of fruit wines. Moreover, defatted sea buckthorn juice can be used as an additive to wheat beer to enhance its taste, nutrition, and colour. In the new study, Belcar et al. [135] explored the possibility of adding defatted sea buckthorn juice to wheat beer as an enhancer. The reported results indicated that the addition of 5% defatted sea buckthorn juice resulted in wheat beer with better colour, higher polyphenol content, and antioxidant capacity.

Improving the organoleptic indices and physicochemical properties of sea buckthorn alcohol is critical for the sea buckthorn alcohol industry to achieve high-quality processing. This can be achieved by analyzing scent, colour, and nutrient contents. Analysis of microbial metabolic activity during the fermentation process to better understand the mechanisms involved. Moreover, the aroma of sea buckthorn alcohol can be enhanced through the utilisation of clarification treatment technology. Skimmed sea buckthorn beverages can be added to improve the colour, nutrient content, and antioxidant activity of sea buckthorn alcohol. All of these methods can achieve superior quality processing of sea buckthorn alcohol.

### 4.4. Sea Buckthorn Oil

As a functional oil, sea buckthorn oil is rich in bioactive components. Sea buckthorn oil can be divided into sea buckthorn pulp oil and sea buckthorn seed oil according to the different extraction sites. Sea buckthorn pulp oil, as a kind of functional fat, is extracted along with the processing of sea buckthorn pulp and has unique fatty acids. Not only is it rich in oleic acid and linoleic acid, but its palmitoleic acid content is significantly higher than that of general vegetable oils [136]. Sea buckthorn seed oil has a higher grease content than sea buckthorn pulp oil. Linoleic acid, linolenic acid, and oleic acid are the primary components in sea buckthorn oil. Unsaturated fatty acids in sea buckthorn oil are helpful in alleviating cardiovascular disease and lowering blood cholesterol levels.

Although sea buckthorn oil is rich in nutritional value, the problem of low oil yield has profoundly limited the industrial development of sea buckthorn oil [137]. Up to now, the extraction methods of sea buckthorn oil include pressing, organic solvent extraction, supercritical CO_2_ extraction, water substitution, the water enzyme method, and ultrasonic and microwave-assisted extraction [19,138,139].

However, these methods are not the best way to achieve superior quality in sea buckthorn oil products. The pressing method reduces the quality of sea buckthorn oil due to the increase in internal friction and temperature caused by squeezing. The organic solvent extraction method has the problem of solvent residuals. Although supercritical CO_2_ extraction has a high extraction rate and can efficiently retain bioactive components, the equipment space is limited, the cost is high, and the energy consumption is high. Hence, it is not appropriate for large-scale industrial applications. The water-generation method will destroy the unsaturated fatty acids in sea buckthorn oil. The water enzyme approach causes emulsification during the extraction process, and the cost is high, making it difficult to utilise in industrial production. Organic solvent extraction and supercritical CO_2_ extraction are frequently coupled with ultrasonic and microwave-assisted extraction methods [140]. It can reduce the extraction time and enhance the quality of oil, but it is not widely used in industry.

In this context, producing high-quality sea buckthorn oil involves exploring new extraction processes or optimising existing methods while taking cost into account. While researchers have utilised subcritical extraction techniques to obtain rice bran oil [141], no relevant studies have been conducted on sea buckthorn oil. The subcritical extraction technique is environmentally friendly, with a low extraction temperature and pressure and a short time, which protects the extracted compounds better. It can draw on other substances’ subcritical extraction techniques to extract sea buckthorn oil and accomplish high-quality processing.

### 4.5. Sea Buckthorn Pomace

The pomace of sea buckthorn is the residue of the beverage used for juice extraction and processing, accounting for 20% of the entire fruit weight. It contains abundant nutrients, including carotenoids, polyphenols, and fatty acids [142], etc. As a by-product of sea buckthorn pulp, it is generally wasted during processing and is underutilised.

The use of sea buckthorn pomace is diverse. It can be ground into a powder and mixed into other foods, or the flavonoid can be extracted to maximise resource utilisation. Stanciu et al. [143] added different concentrations of sea buckthorn pomace powder to flour to explore its effect on the structural, nutritional, and organoleptic properties of bread. The findings suggested that the addition of 8% powdered sea buckthorn pomace yielded the finest organoleptic qualities. It contains large amounts of fibre, protein, and lipids. It has strong antimicrobial and antioxidant properties. Tian et al. [144] optimised the extraction process of total flavonoid from sea buckthorn using the pectinase synergistic ultrasonic method. The optimal extraction technique was as follows: pectinase addition of 5.1%, liquid-to-feed ratio of 41:1, ultrasonic extraction period of 81 min, and total flavonoid extraction of 8.91 mg/g. Meanwhile, sea buckthorn oil can be extracted from the pomace of sea buckthorn. The product quality of sea buckthorn seed oil from sea buckthorn pomace would be affected by different pretreatment and drying techniques.

Two factors must be considered when attempting to achieve high-quality processing of sea buckthorn pomace. To begin, businesses should use sea buckthorn pomace to create a complete industrial chain and maximise resource utilisation. The second is to explore new process routes or improve the current process to achieve large-scale production according to the different processing techniques of sea buckthorn dregs.

## 5. Conclusions and Recommendations

This review provides a comprehensive analysis of the current state of sea buckthorn drying and high-quality processing. It provides an overview of the effects of various pretreatment and drying processes on the drying properties and quality of sea buckthorn, as well as the current issues with high-quality sea buckthorn processing. It also indicates the approaches to attaining high-quality processing of sea buckthorn products. In view of the current problems of sea buckthorn drying and high-quality processing, the following three directions of development can be considered for the future:

(1) Throughout the drying process of sea buckthorn, the drying rate plays a crucial role in reducing product quality maintainance and energy consumption. The application of pretreatment technology prior to sea buckthorn drying is a significant step to enhance the drying rate and quality and decrease energy consumption. According to the research, combined pretreatment can integrate the advantages of the two pretreatments, while reducing costs and improving drying quality and efficiency. In the future, we should concentrate on the use of integrated pretreatment methods in conjunction with the actual sea buckthorn drying requirements. A balance between economy, ecology, and efficiency should be found after recognising the gap between laboratory research and practical manufacturing.
(2) When processing dried sea buckthorn pulp, some small- and medium-sized enterprises (SMEs) prefer natural drying as a cost-effective processing method. Although this process is advantageous to businesses, it has significantly reduced the nutrients in sea buckthorn pulp and cannot bring forth the true worth of sea buckthorn pulp. While hot-air drying and infrared-radiation drying are faster than natural drying, they can cause greater deterioration in quality. Pulsed vacuum drying and vacuum freeze-drying are considered to have superior drying quality but suffer from slow drying rates and excessive energy consumption. In the actual production of sea buckthorn pulp drying, except for vacuum freeze-drying for the processing of sea buckthorn freeze-dried powder and natural sun-drying for the production of sea buckthorn dried fruit, the rest of the drying technology is not widely used. In this situation, it is very necessary for us to start with the research and development of drying equipment or sea buckthorn itself. Firstly, new online-measurement and control technology is introduced into the drying equipment. LF-NMR technology is used to monitor the moisture migration information, predict the drying endpoint to speed up the drying rate, and save energy consumption. The second is to study the best pretreatment technology in the early stage under different drying methods. Pretreatment technology is used to speed up the drying rate, saving energy consumption and reducing the quality deterioration at the same time. Thirdly, research on new drying technology of sea buckthorn is carried out. Through the basic theoretical research on sea buckthorn drying, the macroscopic changes in quality were investigated from the microscopic level. The effects of different drying methods and pretreatment methods on the drying characteristics and quality change mechanism of sea buckthorn were investigated. It is important to investigate the effect of different drying methods and pretreatment methods on the drying characteristics and quality change mechanism of sea buckthorn, which will be useful for the research of new pretreatment technologies such as high-pressure processing (HPP), pulsed electric field (PEF) and new drying technologies such as low-pressure superheated steam drying (SSD), far-infrared-radiation-heating-assisted pulsed vacuum drying. Radiation heating combined with pulsed vacuum drying (FIR-PVD), hot-air combined with vacuum freeze-drying (HAD-VFD), and far-infrared-radiation heating combined with pulsed vacuum drying (FIR-PVD) were applied to sea buckthorn pulp drying with significant promotion.
(3) To fully maximise the product value of sea buckthorn pulp, high-quality processing of sea buckthorn goods must begin at the front end of processing. From beverage to pomace, a complete industrial chain needs to be formed to maximise the use of resources. On this basis, new process routes should be explored or existing processes optimised in combination with the actual situation. It is also necessary to develop the deep-processing industry for sea buckthorn and improve the added value of sea buckthorn processing products. Using the above methods, the sea buckthorn processing industry will realise high-quality production.

## Data Availability

The datasets generated for this study are available upon request from the corresponding author.
